# Kinematic Locomotion Changes in C57BL/6 Mice Infected with *Toxoplasma* Strain ME49

**DOI:** 10.3390/microorganisms7110573

**Published:** 2019-11-18

**Authors:** María de la Luz Galván-Ramírez, Angel Gustavo Salas-Lais, Sergio Horacio Dueñas-Jiménez, Gerardo Mendizabal-Ruiz, Ramón Franco Topete, Sofía Citlalli Berumen-Solís, Laura Roció Rodríguez Pérez, Karina Franco Topete

**Affiliations:** 1 Laboratorio de Neurofisiología, Centro Universitario de Ciencias de la Salud, Universidad de Guadalajara, Sierra Mojada 950, col.Indepdencia, Guadalajara 44320, Mexico; 2 Instituto of Oftalmología Conde de Valenciana I.A.P.”, Chimalpopoca 14, Centro, Ciudad de México 06800, Mexico; 3 Departamento de Ciencias computacionales, Centro Universitario de Ciencias Exactas e Ingenierías, Universidad de Guadalajara, Blvd. Gral. Marcelino García Barragán 1421, Olímpica, Guadalajara 44430, Mexico; 4 Departamento de Microbiología y Patología, Centro Universitario de Ciencias de la Salud, Universidad de Guadalajara, Sierra Mojada 950, col.Indepdencia, Guadalajara 44320, Mexico; 5 Departamento de Anatomía Patológica del Nuevo Hospital Civil de Guadalajara, Salvador Quevedo y Zubieta 750, Independencia Oriente, Guadalajara 44340, Mexico

**Keywords:** *Toxoplasma* infection, locomotion, kinematic, acute, chronic, mice

## Abstract

Chronic infection with the intracellular parasite *Toxoplasma gondii* produces an accumulation of cysts in the brain and muscle, causing tissue damage. The cysts in the brain motor regions affect some kinematic locomotion parameters in the host. To localize the brain cysts from *Toxoplasma gondii* and study the changes in kinematic locomotion in C57BL/6 mice. Female adult C57BL/6 mice were infected orally with 30 ME-49 *Toxoplasma gondii* cysts. An uninfected group (*n* = 7) and two infected groups, examined 15 and 40 days postinfection, were used for this study. To evaluate kinematic locomotion, the mice were marked with indelible ink on the iliac crest, hip, knee, ankle, and phalangeal metatarsus of the left and right hindlimbs. At least three recordings were carried out to obtain videos of the left and right hindlimbs. Mice were video recorded at 90 fps at a resolution of 640 × 480 pixels while walking freely in a transparent Plexiglass tunnel. We measured the hindlimb pendular movement and the hindlimb transfer [linear displacement] curves for each step and evaluated them statistically with Fréchet dissimilarity tests. Afterward, the mice were sacrificed, and the brain, heart, skeletal muscle, lung, liver, and kidney were obtained. The different tissues were stained with hematoxylin and eosin for analysis with optical microscopy. Topographic localization of the cysts was made using bregma coordinates for the mouse brain. The cysts were distributed in several brain regions. In one mouse, cyst accumulation occurred in the hippocampus, coinciding with an alteration in foot displacement. The step length was different among the different studied groups.

## 1. Introduction

*Toxoplasma gondii* (*T. gondii*) is an obligate intracellular protozoan parasite that infects both humans and animals. *Toxoplasma* is transmitted orally, through a transplacental pathway or blood transfusion or via transplant [1]. A third of the world’s population has been affected by this parasite.

*T. gondii* reproduces sexually in the small intestine of the cat, forming immature oocysts that are eliminated in the cat’s feces. Once mature, it is highly infectious via contaminated food ingestion. The asexual reproductive cycle of *T. gondii* is binary, forming tachyzoites. Chronic infection causes the formation of cysts in the muscle and brain.

The forms of bradyzoites inside a tissue cyst are important in the life cycle and for the transmission of toxoplasmosis. In immunocompetent organisms, tissue cysts predominate during chronic infection; however, these cysts are produced early in the infection. In mice, tissue cysts form 2 or 3 days after the parenteral inoculation of tachyzoites. The formation of bradyzoites is after the oral ingestion of oocysts. The tissue cysts form between 5 and 6 days after the ingestion of the bradyzoites.

Once formed, tissue cysts persist for a long time, perhaps throughout the life of the host. It has been suggested that an occasional rupture of the tissue cysts occurs, releasing the bradyzoites. The location and number of tissue cysts in animals differ depending on the strain of *T. gondii*.

Several authors have shown that chronic toxoplasmosis in mammals causes deficits such as learning alterations, loss of sensory attention, impaired spatial memory, motor performance, and increased anxiety [2,3,4,5].

Previous studies have reported heterogeneous cyst distribution in the mouse brain. Cysts were found in the olfactory bulb, entorhinal, somatosensory, motor, orbital, frontal and visual cortices. Cysts were also found in the hippocampus and in the amygdala, which could be related to behavioral alterations. *Toxoplasma gondii* also produces a significant deficit in motor coordination, balance, and gait [6,7]. Brain histopathological lesions are *Toxoplasma* strain-dependent. It was observed 18 weeks postinfection that cysts were distributed throughout the brain without selective tropism [2]. They also produced a sensorimotor deficit in C57BL/6 mice infected with the ME-49 *Toxoplasma* strain: the mean stride length and the number of rear paw drags were significantly different between *T. gondii*-infected and control mice [8]. It has been reported that animals with brain cysts reach higher locomotion speeds and have an initial acceleration during the first second of movement that is twice that of control groups [9]. It is of great interest to study different kinematic parameters, such as articular angular changes, stride length, limb linear to displacement and histological changes in several tissues.

Histopathology in the brains of mice infected with *Toxoplasma gondii* shows endothelial hypertrophy and gliosis [10]. The *T. gondii* ME-49 strain produces cortical and subcortical lesions. The most prominent pathological change was cellularity due to transmigration of peripheral inflammatory cells to the brain. Cyst presence produces parenchymal hemorrhage, edema, and limited degeneration of the cortical neurons.

In the spinal cord, histopathological examinations revealed that lesions are similar to those observed in the brain, with the presence of many infiltrated inflammatory cells [11].

Infection of skeletal muscle cells by *T. gondii* is due to the differentiation from the highly replicative tachyzoites to dormant bradyzoites, and tissue cyst formation is needed for parasite persistence in muscle tissue [12].

Experimentally, in mice infected with *Toxoplasma*, changes in the muscular capillaries including loss of the endothelial wall, occlusion of the lumen, and necrosis have been observed. Motor endplates are abnormal, muscle fiber atrophy varies from light to severe, and endplates show terminal axon damage and modified postsynaptic regions [13]. The described damage can affect host locomotion.

The purpose of this study is to quantitatively establish kinematic alterations in mice acutely and chronically infected with the ME-49 strain of *Toxoplasma*. The second purpose will be histopathological analysis was also performed in the brain, liver, lung and muscle tissues to study the damage in these tissues. The damage of different organs as: heart, skeletal muscle, lungs, and other tissues can be implied in locomotion alterations.

## 2. Material and Methods

### 2.1. Ethical Aspects

This study was carried out in strict accordance with the recommendations of Official Mexican Standards NOM-067. The protocol was approved with registration number CI-07618. All animal experiments were approved by the biosafety, research, and ethics committees of the University Center of Health Sciences of the University of Guadalajara, with number CUCS/CINV/0457/18, 11 December 2018.

### 2.2. Cysts

Female mice C57BL/6 (wt) of 8–10 weeks of age, were fed ad libitum, under conditions of 12 h light/dark in the bioterium of the Ophthalmology Institute “Foundation of Private Assistance Count of Valenciana I.A.P. Cysts were obtained from oral infected mice at 42 days post-infection. The mice were anesthetized with pentobarbital, intraperitoneally, and then sacrificed. Cysts were harvested from infected brain mice. The brain tissues were homogenized and washed twice with PBS, and low virulence cysts of *Toxoplasma gondii* were obtained, which were used to infect all the experimental mice.

### 2.3. Experimental Groups

All experiments were conducted in eight-week-old, adult, female C57BL/6 mice obtained from the Institute of Ophthalmology Conde de Valenciana I.A.P., México City, México. Three groups of mice were studied: Groups 1 and 2 were orally infected with a dose of 30 cysts. In Group 1 (*N* = 7), the mice were sacrificed 15 days postinfection and were named the acute-infected group. In Group 2 (*N* = 12), the mice were sacrificed 40 days postinfection and were named chronic-infected group. Group 3 (*N* = 7) was the uninfected mice (control group).

### 2.4. Locomotion Studies

**Figure 1:**
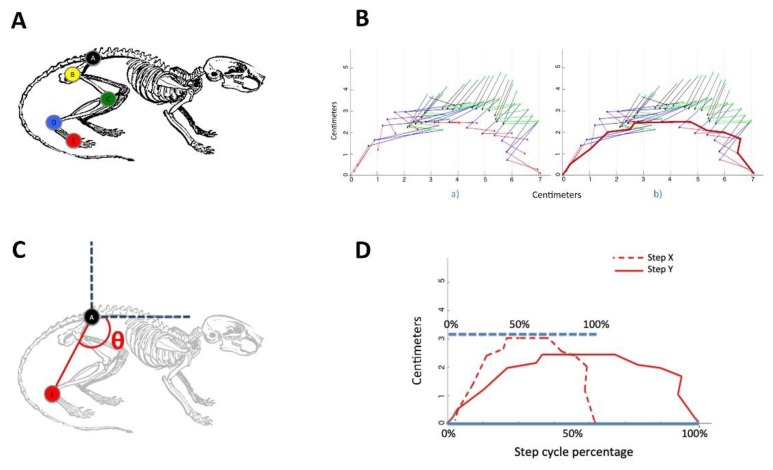
Method to evaluate different kinematic locomotion parametres. (**A**) Selected hindlimb joints for locomotion pattern analysis. Control, chronic and acute mice were marked at five points on the left and right hindlimbs using a nontoxic marker. (**B**) The different color lines illustrated different lines segments between two market points. (a) An example of the pattern generated by the transition phase of a mouse, and (b) the curve corresponding to the amplitude of point E across time. (**C**) Depiction of the measured angle employed for the hindlimb pendulum-like movement analysis. (**D**) Depiction of two normalized curve patterns with respect to the step cycle.

All mice from the three groups were marked with nontoxic indelible ink in the (A) iliac crest, (B) the hip, (C) the knee, (D) the ankle, and (E) the phalangeal metatarsus, in both the left and right hindlimbs (Figure 1A). Mice were video recorded at 90 fps at a resolution of 640 x 480 pixels while walking freely in a transparent Plexiglass tunnel [14,15].

The marks on the mice were manually annotated on each frame of the videos using proprietary software developed by our research group, and marks were used to track their displacement in the horizontal and vertical directions during mouse movements (Figure 1B). The duration and covered distance of the translation phase for all steps for each group were computed. Step length was divided and evaluated in pixels. Then, transformed into centimeters and plotted in the ordinate, the different mice groups were plotted on the abscissa.

The translation phase patterns of the marks of all mice were compared by two means: the analysis of the pendulum-like movement [16,17] that is measured as the curve corresponding to the changes in the angle resulting from connecting points A and E with respect to time (Figure 1C), and the analysis of the joint transitions that was performed by computing curves corresponding to the amplitude of the joints movement in the horizontal and vertical directions across time (Figure 1B).

Steps length and speed can be different among mice. Therefore, to evaluate the joints, motion patterns were necessary to normalize the curves. We divided the steps using 20 points at equal time bins during the step cycle. The beginning of the translation phase corresponded to the 0% step cycle, and the point was corresponding to the end of the translation phase corresponded to 100% of the step cycle (Figure 1D).

Statistics for all normalized curves corresponding to each mark of the control group mice were computed for analyses. Then, each bin corresponding to chronic- and acute-infected mice was compared with respect to the statistics of the control curves [15,16].

### 2.5. Statistical Analysis

To validate the data normality we used the Kolmogorov-Smirnov Test. Our data are normally distributed (p-value of 0.08). Then, we performed a t-test with a 95% confidence level (α = 0.05) to determine if statistically significant differences existed between the control vs chronic and control vs acute groups [15].

### 2.6. Histopathological Samples

Mice infected and remaining alive for 15 (*N* = 7) days and those surviving for 40 (*N* = 12) days, as well as mice in the control group (*N* = 7), were anesthetized with isofluorane and sacrificed. Subsequently, a craniotomy of the mouse was performed to extract the brain, while other organs such as the heart, skeletal muscle, lung, liver, and kidney were also extracted; they were washed with saline solution before formol fixation. Then, the brains were placed in tubes containing PBS at 10% until the sample was covered. Twenty-four hours later, samples were washed with distilled water and then fixed. PBS was replaced with 70% alcohol. Samples were embedded in paraffin to obtain 0.5-micron slices [6].

### 2.7. Staining with Hematoxylin and Eosin

The slides were placed in a hematoxylin container (Sigma Aldrich, Darmstadt, Germany) for two minutes, and then, the excess was removed with distilled water. The slices were submerged for a three-second period in acid alcohol (1% HCl in 70% alcohol), washed with water, and then immersed in yellow eosin (Sigma) for a minute and a half before being washed with tap water for thirty seconds. For dehydration, passages were made in increasing gradients of alcohol and xylol as follows: 70% alcohol for 3 s, 90% alcohol for 3 s, 96% alcohol for 3 min, twice in 100% alcohol for 5 min, and then twice in xylol for 5 min. Finally, combined with entellan, the obtained cuts were observed under a microscope (Carl-Zeiss Oberkochen, Germany) at 10 X and 40 X.

The Histological analysis was qualitative. We name four categories (Absent, Mild, Moderate, and Severe). The categories were established according to the morphological severity damage and the number of affected cases. The topographical location of cysts in the brains of infected mice was localizated using the bregma coordinates of the mouse brain [18].

## 3. Results

### 3.1. Step and Displacement Analysis

**Figure 2:**
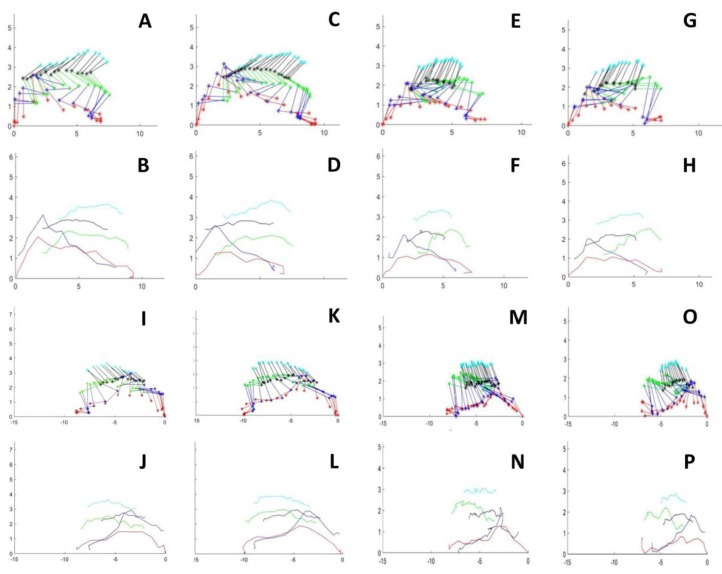
Stick figures of two consecutive steps of the right and left hindlimbs in control and an acute infected mice. Mouse walking from right o lefth an viceverse. (**A**,**C**) Two steps of right hindlimb walking from lefth to right in a control mouse. (**E**,**G**) Two steps of right hindlimb walking from lefth to right in an acute-infected mouse. (**I**,**K**) Two steps of lefth hindlimb walking from lefth to right in a control mouse. (**M**,**O**) Two steps of lefth hindlimb walking from lefth to right in an acute infected mouse. (**B**,**D**) and (**F**,**H**) illustrates the linear hindlimb displacement for each marked point in control and in an acute-mice, respectively. (**J**,**L**) and (**N**,**P**). Illustrates the linear hindlimb displacement for each marked point in control and in an acute-mice, respectively. Ordenates and abscissas for all graphs are in centimeters.

**Figure 3:**
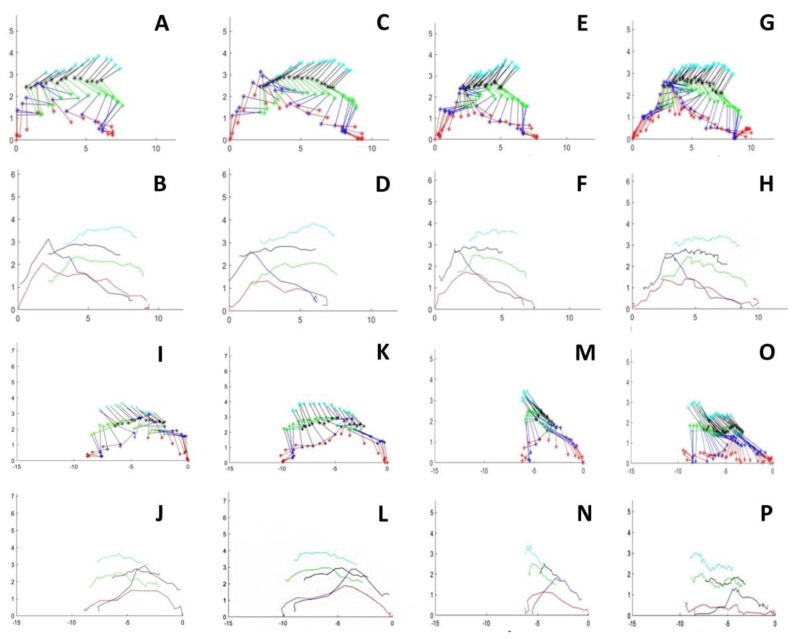
Stick figures of two consecutive steps of the right and left hindlimbs in control and an chronic infected mice. Mouse walking from right o left and viceversa. (**A**,**C**) Two steps of right hindlimb walking from left to right in a control mouse. (**E**,**G**) Two steps of right hindlimb walking from left to right in an acute-infected mouse. (**I**,**K**) Two steps of left hindlimb walking from left to right in a control mouse. (**M**,**O**). Two steps of left hindlimb walking from left to right in an acute infected mouse. (**B**,**D**) and (**F**,**H**) illustrates the linear hindlimb displacement for each marked point in control and in an acute-mice, respectively. (**J**,**L**) and (**N**,**P**) Illustrates the linear hindlimb displacement for each marked point in control and in an acute-mice, respectively. Ordenates and abscissas for all graphs are in cm.

There were no significant differences among the studied groups in pendular movement (not shown). In contrast, the results indicated a statistically significant difference in the step length of control vs chronically infected mice, as well as between the control and acute groups in the left and right hindlimbs (Figure 2 and Figure 3).

**Figure 4:**
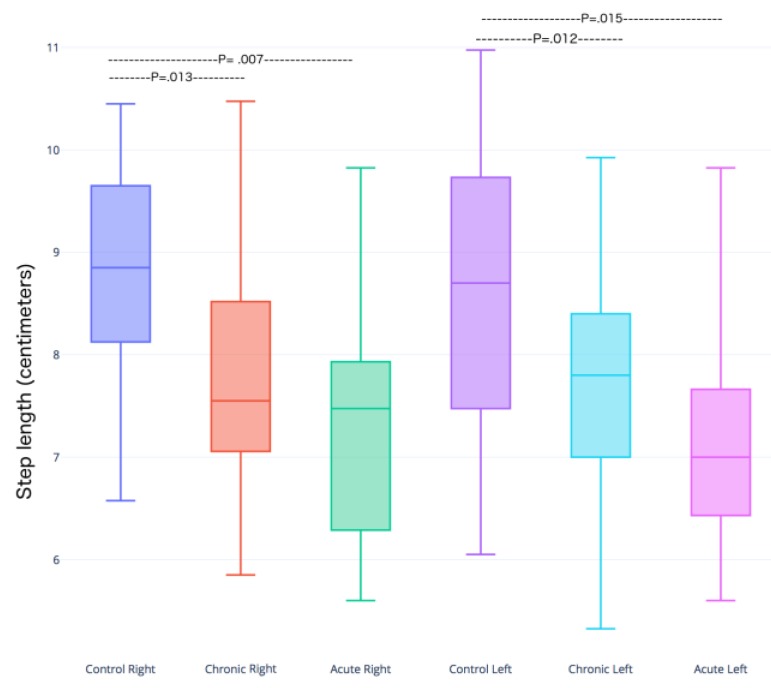
Box plots depicting the distribution of the step lengths in control, acute and chronic infected mice groups. The ordinate for each group for the right and left hindlimbs. The p-value of the Student’s t-test (α = 0.05) for each group is reported in the graph. There is greater change in step length in the right leg in the acute infection group vs the control; the difference in the left step length was significant, *p* < 0.015.

In the box plot graphics, 75% of the data are placed in each box, and the median and its respective standard deviations are calculated and platted. Statistically significant differences existed between the control vs chronic and control vs acute groups (Figure 4).

**Figure 5:**
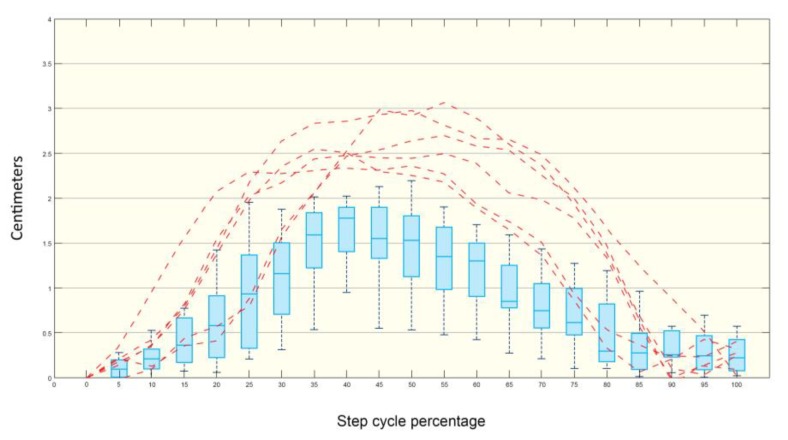
Control mice metatarsal joint displacement in boxplots. Dashed lines indicate a qualitative comparison between control and mouse 7. The red lines show the dissimilarity existing between the cycle percentages in the mice of the acute-infected group compared to the control group.

The curves for linear hindlimb displacement in infected mice were similar among the control and experimental groups. However, there was a relevant difference in the pattern of the curve for the foot displacement of mouse 7 in the chronic group (Figure 5). Note that the amplitude of the metatarsal joint displacement (lifting up) curves was out of range, more extensive than that of the control group, as depicted in the box plots.

### 3.2. Brain Toxoplasma Cyst Distribution

**Figure 6:**
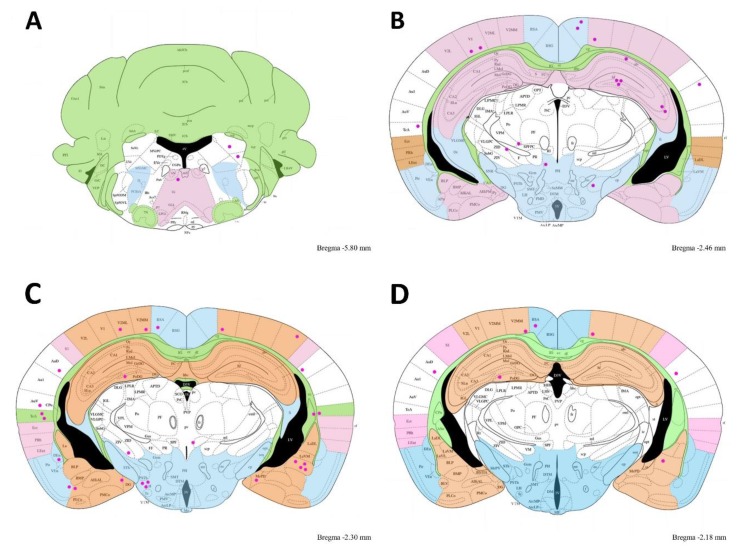
Brain schematic representing the cyst distribution in the mouse 7 from bregma −1.94 to 1.34. The cuts are from a bregma distance indicated in the Figure in small leter in the right corner of each cut. Cysts are pink dots. Note cyst groupings found in the hippocampus, temporal and auditory cortex, amygdaline nucleus and hypothalamus.

The brain cyst distribution for this particular mouse is illustrated in Figure 6. *T. gondii* cysts were located in groups of three to four cysts in the hippocampus zone in the cuts of bregma −1.94 to 1.34. However, *Toxoplasma* was widely distributed in all brain zones.

**Figure 7:**
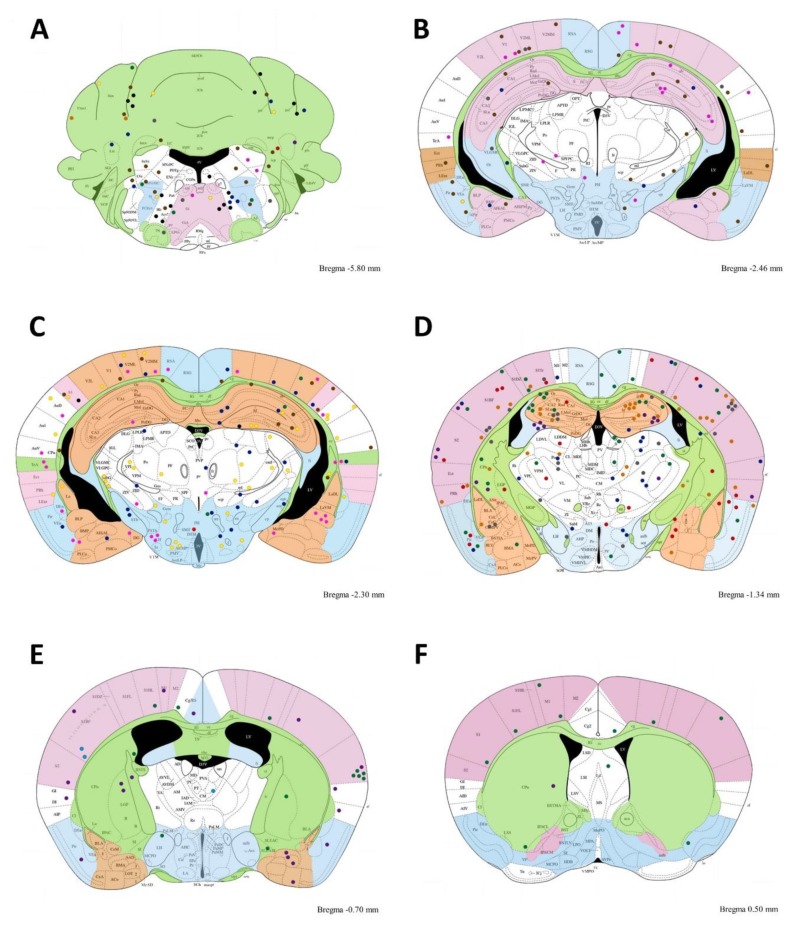
Brain schematic representing the cyst distribution accumulation. Grouping cysts between several rostral cuts in chronic infected mice. *T. gondii* cyst distribution. The figure illustrates several coronal histological cuts from bregma −6.0 to bregma 0.38. Abbreviations indicate the brain zones (see abbreviation list). **A**–**F_A; B; C; D; E; F;_**) Dots indicate brain cyst localization in twelve mice at a bregma distance indicated on the Figure for each cut. (**A**) Grouped cysts from bregma −6.0 to −5.8, (**B**) grouped cysts from −2.54 to −2.46, (**C**) grouped cysts from −2.06 to −2.30, (**D**) grouped cysts from −1.94 to −1.34, (**E**) grouped cysts from −0.94 to −0.70, and (**F**) grouped cysts from 0.38 to 0.50. Note in A there is cyst accumulation in Acs7, Irt, and Pa6. Additionally, in (**D**), there is cyst accumulation in the hippocampal zones and SIBF zone; (**E**) shows cyst accumulations in S2. All cysts pink color correspond to those illustrate in Figure 6.

In all mice, cysts were widely distributed in the brain. We analyzed several brain coronal cuts to observe the distribution of *T. gondii* cysts. The cuts were made from bregma −6.0 to 0.38. In general, the cysts were localized randomly in several brain zones, as illustrated in Figure 7.

*Toxoplasma* cysts were widely distributed in all brain regions, as illustrated in Table 1. However, some brain zones (hf, LH, PoDG, RSA, SIBF, S2, TeA, V1,ZID) were preferred. In these zones there were more than ten cysts per region.

### 3.3. Cysts in Acute Mice

In acute infected mice we only observed eighteen cysts (most of them in primary motor cortex, (*N* = 5), three in caudate putamen. Ten cysts were distributed in six brain regions.

### 3.4. Histological Analysis

**Figure 8:**
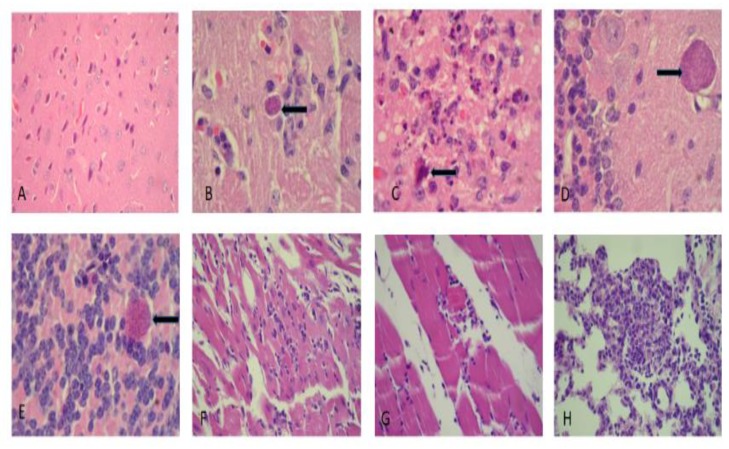
Histological cuts from brain, cardiac muscle and lung in the acute-infected group. (**A**) Brain cortex illustrating neuronal hypoxia (400×), (**B**) gliosis with a *Toxoplasma gondii* bradyzoite cyst (400×), (**C**) site exhibiting glionecrosis with a neuron in apoptosis (1000×), (**D**,**E**) *Toxoplasma* cyst placed in the cerebellar molecular and the granular layers (1000×), (**F**) moderate myocarditis (400×), and (**G**) moderate myositis with a fragmented muscle fiber (400×), (**H**) focal pneumonitis with acute and chronic components (400×). The arrows indicate *Toxoplasma* cysts except in figure C wich indicates an apoptotic cell.

A microscopic analysis of the mouse tissue was examined and showed a preferential tropism of *Toxoplasma gondii* in the central nervous system. In the acute infection group, gliosis nodules with remarkable necrosis accompanied by few *Toxoplasma* cysts either inside or outside of the gliosis were observed. We observed a neuronal hypoxia booth in cortical and hippocampal regions, with a less prominent meningovascular inflammatory component. The cysts were also found in the cerebellum. In the chronically infected mouse group, mild- to moderate-intensity myocarditis was found in most animals, with myopathy in 20% of cases; lymphocytic myopathy was the predominant type. We also noted pulmonary disease characterized by patches of a mixed inflammatory infiltrate with lymphocytes macrophages and polymorphonuclear leukocytes. A mild portal-reactive hepatitis was found in the liver, and in one case, mononuclear focal inflammation was found in the renal parenchyma (Figure 8).

**Figure 9:**
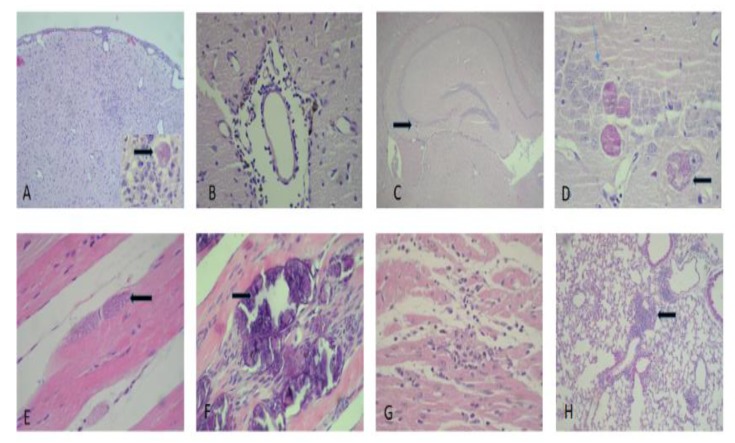
Histological cuts from brain cortex, skeletal muscle and lung in the chronic-infected group. (**A**) Brain cortex with meningitis, gliosis (100×) and a cyst (1000×). (**B**) Lymphocytic perivascular inflammation accompanied by an infiltrate (400×). (**C**) Hippocampus with cysts (100×). (**D**) A cyst illustrating a rejection of the neuronal nucleus (thick blue arrow; 1000×). (**E**) *Toxoplasma* cysts in the skeletal muscle sarcoplasm (400×). (**F**) Dystrophic calcification in the sarcoplasm due to cyst destruction. Note adjacent tissue regeneration (1000×). (**G**) Moderate myocarditis (400×). (**H**) Inflammatory pulmonary infiltrate; predominantly peribronchiolar (100×). The arrows indicate *Toxoplasma* cysts.

In the chronically infect mouse group, we also observed more brain cysts compared to the acute experimental group. Cyst distribution appeared in several brain regions from the metencephalon to the telencephalon with an accumulation in the hippocampus. Gliosis was variable, with a tendency to be present in the cortical and periventricular regions. Mild to severe meningitis was accompanied by parenchymal perivasculitis and included endothelial proliferation (Figure 9).

In six out of 12 cases, skeletal muscle damage occurred. Damage included mild to focal myositis. There was evidence of cysts in the sarcoplasm in one case. In four out of 12 cases there was dystrophic calcification of muscle fibers that ranged from moderate or severe. Mild to moderate chronic inflammation was observed in the lungs in both the peribronchiolar and parenchymal tissue. Focal pneumonitis with a chronic component was also observed (Figure 9, Table 2).

## 4. Discussion

We did not find pendulum angular movement changes among the different study groups. Angular changes in the different hindlimb joints could compensate for the absence of change in the pendular movement. Further studies using camera recordings at 250 frames/sec will be done to clarify this point. In contrast, we observed alterations in the stride length as found by other authors [8]. We did not study rear paw drags. The metatarsial joint displacement curve was different in one chronically infected mouse compared to the control group. In this mouse, we observed cyst accumulation in the hippocampus, which can alter the locomotion kinematics. In rats, penetrating and damaging the hippocampus produces an alteration in locomotion speed [19].

We did not study whether the hippocampus cysts had enough time to liberate the bradyzoites to produce the kinematic alterations in this particular mouse. Note the clear neuron damage and hippocampal cysts in Figure 9C,D. The method used in these experiments to explore locomotion kinematics is reliable enough to detect changes in a joint through linear displacement curves.Traumatic brain injure in rats and mice hippocampus produce locomotion velocitiy reduction [17,19]. In this study most of cysts were found in a mouse with clearly change in join metatharsal displacement.

A sensorimotor deficit in C57BL/6 mice infected with the ME-49 *Toxoplasma* strain was also reported previously [8]. Gait deficits, measured as the number of missteps, the mean stride length and the number of rear paw drags, were significantly different between *T. gondii*-infected and control mice, *p* < 0.05 [8].

In an open field locomotion study of female C57BL/6 mice infected with transgenic *Toxoplasma gondii* ME49, it was found that the animals with brain cysts reached higher locomotion speeds in as early as the first second of locomotion and showed an initial acceleration during that first second of movement that was double that of control groups [9]. We did not study mice in open field locomotion to compare to the results of Alfonso et al. [9]. It is our intention to perform further studies on mice in open field locomotion using several recording cameras to study several kinematic locomotion parameters. For the first time, we implemented an analysis of iliac crest, hip, and ankle joint translation and a computational program to evaluate statistical significance among curves generated in different mouse groups using a Fréchet dissimilarity analysis. This method will be used in further experiments to statistical evaluate join displacement [20,21].

Acute infected mice had neuronal gliosis and necrosis damage, as well as an apparent cyst breakdown with consequent bradyzoites liberation, which changed them to chronic status and produced meningeal-encephalitic damage [8]. Neurons, astrocytes, and microglia are damaged by the presence of cysts in the neuronal body and in the dendrites and axons [22,23]. We found cysts in damaged neurons.

The cysts in chronic infected mice were distributed in several brain regions: mainly hippocampus, and the telencephalons.A minor number of cysts appeared in the brain stem and cerebellum. In acute infected mice we observed few cysts than in chronic infected mice, distribuited in several regions, manly in motor cortex. Given the distribution, several brain functions could be affected. It was also suggested that the presence of brain cysts was due to local damage in the hamate-encephalic brain barrier or alterations in metabolic blood flow [6]. It was suggested that the intercellular adhesion molecule 1 (ICAM-1) is upregulated in cellular barriers during *Toxoplasma* infection. Soluble human ICAM-1 and ICAM-1 antibodies inhibit the transmigration of parasites across cellular barriers, implicating this receptor in the process of transmigration.

Furthermore, an intercellular adhesion molecule (ICAM-1) immunoprecipitated the mature form of the parasite and adhesion microneme protein (MIC2) present on the parasite surface, indicating that this interaction may contribute to cellular migration [24,25] We did not study the intracellular adhesion molecules, but the *Toxoplasma* was widely distributed in the brain, suggesting that the brain barrier had broken down. The present results also demonstrated a disperse distribution of brain cysts, although there was an apparent predominance in the hippocampus. It is important to mention that in acute-infected mice, there was no histopathological damage in tissues such as the lung, liver, heart, and kidney, but damage was present in the chronically infected mice.

Our results suggest the importance of the possible presence of cysts liberating bradyzoites. The presence of cysts in the neural cell body, bradyzoites liberation, and muscle calcification in chronically infected mice produced skeletal muscle damage as well as mild to moderate focal myositis and dystrophic calcification of muscle fibers. This damage has been reported in *Toxoplasma*-infected mice and increases between days 12 and 30 postinfection [15,25]; we believe that this damage in the skeletal muscle reduced the locomotion of the host.

## 5. Conclusions

This study contributes with relevant information that increases knowledge in relation to the damage produced by *Toxoplasma gondii* in several tissues as well as a quantitative analysis of kinematic locomotion.

## Figures and Tables

**Table 1:** Distribution of *Toxoplasma* cysts in differents brain areas in chronic infected mice.

Abbreviation Name	Left	Right	Total
3V 3rd ventricle	1		1
AAD anterior amygdaloid area, dorsal part	3		3
ACo anterior cortical amygdaloid nucleus		1	1
AHiAL amygdalohippocampal area, anterolateral part	3		3
AIP agranular insular cortex		1	1
alv alveus of the hippocampus		2	2
APTD anterior pretectal nucleus, dorsal part		1	1
ArcLP arcuate hypothalamic nucleus, lateroposterior part	3	2	5
Au1 primary auditory cortex	3	1	4
BLP basolateral amygdaloid nucleus, posterior part	1		1
BMA basomedial amygdaloid nucleus, anterior part	1	1	2
BMP basomedial amygdaloid nucleus, posterior part	1		1
cc corpus callosum	3		3
CA1 field CA1 of hippocampus	4	3	7
CA3 field CA3 of hippocampus	5		5
cg cingulum	1		1
Cg/RS cingulate/retrosplenial cortex		2	2
Cg2 cingulate cortex, area 2	6		6
Cl claustrum	3	3	6
CM central medial thalamic nucleus		1	1
cp cerebral peduncle, basal part	2	6	8
CPu caudate putamen	2	8	10
DEn dorsal endopiriform nucleus	1		1
df dorsal fornix			
DG dentate gyrus	1	1	2
ec external capsule	1		1
FC fasciola cinereum	2		2
fi fimbria of the hippocampus		4	4
fr fasciculus retroflexus	2		2
Gem gemini hypothalamic nucleus	1	2	3
Gi gigantocellular reticular nucleus		1	1
GI granular insular ex	1		1
hf hippocampal fissure	10	7	17
ic internal capsule		1	1
IPAC interstitial nucleus of the posterior limb of the anterior commissure	1		1
LA lateroanterior hypothalamic nucleus		1	1
Ld lambdoid septal zone	1		1
IODM inferior olive, dorsomedial cell group	1	1	2
LEnt lateral entorhinal cortex	1		1
LH lateral hypothalamic area	11	7	18
LPLR lateral posterior thalamic nucleus, laterorostral part	1	1	2
LVe latral vestibular nucleus		1	1
mcPV med amyg, postvent	1		1
MCPC magnocellular nucleus of posterior commissure	2		2
ml medial lemniscus	4	3	7
mt mammillothalamic tract	2	1	3
Or oriens layer of the hippocampus	1	1	2
PF parafascicular thalamic nucleus	2	2	4
PH posterior hypothalamic area		6	6
Po posterior thalamic nuclear group	5	3	8
PoDG polymorph layer of the dentate gyrus	15	12	27
PR prerubral field		1	1
PRh perirhinal cortex	3	3	6
Rad stratum radiatum of the hippocampus		1	1
RMg raphe magnus nu	2		2
RSA retrosplenial agranular cortex	7	4	11
RSG retrosplenial granular cortex	2	2	4
Rt reticular thalamic nucleus	3	3	6
S1 primary somatosensory cortex	3	3	6
SolIM solitary nu interm	1	2	3
S1BF primary somatosensory cortex, barrel field	17	12	29
S1Tr primary somatosensory cortex, trunk region	4	5	9
S2 secondary somatosensory cortex	8	5	13
SLu stratum lucidum, hippocampus	2	5	7
SPF subparafascicular thalamic nucleus	1	1	2
st stria terminalis	1		1
STh subthalamic nucleus	1	4	5
Sub submedius thalamic nucleus	1	1	2
SubG subgeniculate nucleus	3	3	6
SubI subincertal nucleus	1	1	2
TeA temporal association cortex	7	3	10
V1 primary visual cortex	6	5	11
V2L secondary visual cortex, lateral area	2	2	4
V2ML secondary visual cortex, mediolateral area	4	2	6
V2MM secondary visual cortex, mediomedial area	5	4	9
VEn ventral endopiriform nucleus		1	1
VL ventral thalm nu	3	1	4
VLGMC ventral lateral geniculate nucleus, magnocellular part	1		1
VLGPC ventral lateral geniculate nucleus, parvicellular part	1		1
VPL ventral posterolateral thalamic nucleus	5	2	7
VPM ventral posteromedial thalamic nucleus	2	3	5
VRe ventral reuniens thalamic nucleus	1	1	2
TC tuber cinereum area	2		2
ZID zona incerta, dorsal part	5	6	11
Total	213	173	386

**Table 2:** Histological findings in the C57BL/6 mice infected with the ME-49 strain of *Toxoplasma gondii*.

Pathological Damage	Acute Group	Chronic Group
Reactive gliosis	+++	++
Glionecrosis	+++	+
Meningoencephalitis	+	+++
Vasculitis	+	−
Cerebral hypoxia	++	+
Cysts in hippocampal cortical region	+	+++
Cysts in the cerebellar region	++	+
Peribronchial inflammation	++	+++
Acute pneumonitis	++	+
Myocarditis	+	++
Myositis	+	+++
Dystrophic calcification of sarcoplasm	−	++
Cysts in sarcoplasm	−	+
Reactive hepatitis	++	+

The intensity of the damage corresponds to: − (none shown), + (slight), ++ (moderate) and +++ (severe).
